# Upgrading of Extra-Heavy Crude Oils by Dispersed Injection of NiO–PdO/CeO_2±δ_ Nanocatalyst-Based Nanofluids in the Steam

**DOI:** 10.3390/nano9121755

**Published:** 2019-12-10

**Authors:** Oscar E. Medina, Cristina Caro-Vélez, Jaime Gallego, Farid B. Cortés, Sergio H. Lopera, Camilo A. Franco

**Affiliations:** 1 Grupo de Investigación en Fenómenos de Superficie—Michael Polanyi, Departamento de Procesos y Energía, Facultad de Minas, Universidad Nacional de Colombia, Sede Medellín, Medellín 050034, Colombia; oemedinae@unal.edu.co (O.E.M.); fbcortes@unal.edu.co (F.B.C.); 2 Grupo de Yacimientos de Hidrocarburos, Departamento de Procesos y Energía, Facultad de Minas, Universidad Nacional de Colombia, Medellín 050034, Colombia; ccarov@unal.edu.co (C.C.-V.); shlopera@unal.edu.co (S.H.L.); 3 Química de Recursos Energéticos y Medio Ambiente, Instituto de Química, Universidad de Antioquia UdeA, Calle 70 No. 52–21, Medellín 050010, Colombia; andres.gallego@udea.edu.co

**Keywords:** adsorption, asphaltene, steam injection, dispersed injection, nanofluid, resins

## Abstract

The main objective of this study is to evaluate the injection of a dispersed nanocatalyst-based nanofluid in a steam stream for in situ upgrading and oil recovery during a steam injection process. The nanocatalyst was selected through adsorption and thermogravimetric experiments. Two nanoparticles were proposed, ceria nanoparticles (CeO_2±δ_), with and without functionalization with nickel, and palladium oxides (CeNi0.89Pd1.1). Each one was employed for static tests of adsorption and subsequent decomposition using a model solution composed of *n*-C_7_ asphaltenes (A) and resins II (R) separately and for different R:A ratios of 2:8, 1:1, and 8:2. Then, a displacement test consisting of three main stages was successfully developed. At the beginning, steam was injected into the porous media at a temperature of 210 °C, the pore and overburden pressure were fixed at 150 and 800 psi, respectively, and the steam quality was 70%. This was followed by CeNi0.89Pd1.1 dispersed injection in the steam stream. Finally, the treatment was allowed to soak for 12 h, and the steam flooding was carried out again until no more oil production was observed. Among the most relevant results, functionalized nanoparticles achieved higher adsorption of both fractions as well as a lower decomposition temperature. The presence of resins did not affect the amount of asphaltene adsorption over the evaluated materials. The catalytic activity suggests that the increase in resin content promotes a higher conversion in a shorter period of time. Also, for the different steps of the dynamic test, increases of 25% and 42% in oil recovery were obtained for the dispersed injection of the nanofluid in the steam stream and after a soaking time of 12 h, compared with the base curve with only steam injection, respectively. The upgraded crude oil reached an API gravity level of 15.9°, i.e., an increase in 9.0° units in comparison with the untreated extra-heavy crude oil, which represents an increase of 130%. Also, reductions of up to 71% and 85% in the asphaltene content and viscosity were observed.

## 1. Introduction

Steam injection has been the main thermal enhanced oil recovery (TEOR) technique for the exploitation of heavy (HO) and extra-heavy crude oil (EHO) reservoirs in recent decades [1,2]. Nevertheless, high costs [3], steam channeling [4], and no appreciable change in crude oil quality [5] are the difficulties that the conventional steam injection has. To increase the efficiency of the steam injection technology and overcome the problems associated with the transport and refining operations because of the low quality of the crude oils [6], non-condensable gases [7] and chemical agents [8] are injected into the reservoir in parallel or in sequence with steam, showing excellent results in some field cases related mainly to the increase in oil productivity. In this order, the injection of chemicals for in situ upgrading has emerged as a promising solution to produce an oil of higher quality after its interaction with catalysts in the reservoir [9]. This occurs through the decomposition of the heaviest oil components into lighter molecules by means of different reactions such as water–gas shift, steam reforming, methanation, and aquathermolysis, among others, that occur under the pressure and temperature conditions of the steam injection process [10], leading to an increase in the American Petroleum Institute (API) gravity, decreasing the sulphur content [11], and promoting an increase in saturated hydrocarbons [12].

The implementation of catalysts also helps to control the heat front temperature [13], enhance the recovery performance [9,14], and promote the steam quality maintenance [15]. As an important frontier technology, the development of catalysts at the nanoscale has been proposed by several authors as a TEOR additive to improve the efficiency of the processes [16,17]. Different nanoparticles of SiO_2_ [18], Al_2_O_3_ [15], TiO_2_ [19], or rare earth oxides [16,17] have been assessed to increase the rate of gasification reactions under steam injection conditions [20]. These nanoparticles have been used as support nanocatalysts for transition element oxides (TEOS) such as Pd [9,21], Pt [22], Co [16], Ni [23,24], and Fe [25] to improve the adsorption and catalytic decomposition of the oil heavy fractions such as asphaltenes and resins [16,26]. The injection of the nanoparticles into the porous medium has been carried out mainly through batch injection of the nanofluid containing the nanocatalysts [18] or by means of a pre-treatment of the heavy crude oil sample under static conditions [24]. Hashemi et al. [27] reported the implementation of Ni–W–Mo ultra-dispersed nanocatalysts for in situ upgrading of Athabasca bitumen between 320 and 340 °C under a hydrogen flow rate, obtaining increases in API gravity and decreases in the sulfur and nitrogen content favored by the hydrogenation reactions and subsequent coke formation inhibition. As for the batch injection of nanofluid, Cardona et al. [18] used silica nanoparticles with 1.0 wt.% of nickel and palladium oxides at 220 °C for the steam injection test. The authors found an increase from untreated crude oil to upgraded oil in API gravity from 7.2° to 12.1° and reductions in the asphaltene content of 40%. Although the injection of nanofluid in batch-mode showed a good result, the nanofluid injection into the reservoir must be considerably improved to achieve a deeper penetration in the reservoir and a better distribution within the porous spaces, generating a greater interaction with heavy crude oil molecules.

In this way, recently, a novel technique for well stimulation and fluid injection known as gas stimulation (GaStim) was proposed by Franco et al. [28] and Restrepo et al. [29]. The method consists of the dispersion of a liquid treatment on the gas stream, achieving high injection rates and liquid droplets with a smaller size, as well as increasing the invasion radius of the liquid around the injector well [29]. Restrepo et al. [29] reported positive results in the first pilot test in Colombia to overcome the formation damage because of the combined effect of condensate banking and asphaltene precipitation. Through the injection of the treatment by means of the GaStim method, an increase of 40% in oil rate production and a decrease of 30% of the gas–oil ratio was achieved. However, to our knowledge, no studies have reported the dispersed injection of nanofluids for EOR or TEOR applications. With the development of this work, a new approach for the application of nanofluids dispersed in the steam stream and, in general, for future applications in enhanced thermal oil recovery processes is expected.

## 2. Materials and Methods

### 2.1. Materials

An extra-heavy crude oil from a field in the center of Colombia was employed for asphaltene and resin extraction as well as for the displacement tests. The selected EHO has an API gravity of 6.4°, resin and asphaltene contents of 52.0 wt.% and 28.7 wt.%, respectively, and a viscosity of 6 × 10^6^ cP at 25 °C. *n*-Heptane (99%, Sigma-Aldrich, St. Louis, MO, USA) was used to isolate the asphaltenes from the EHO, and chromatographic silica (Sigma-Aldrich, St. Louis, MO, USA) was added to the deasphalted crude oil to separate the resins from the solution. The extraction procedure has been explained in previous studies [30]. The compositions of the isolated asphaltenes and resins are shown in Table 1. The concentrations of heteroatoms in the organic fractions were obtained by elemental analysis using the ASTM D 5291 standard employing an elemental analyzer Flash EA 1112 (Thermo Finnigan, Milan, Italy). The oxygen content was calculated by the difference. Besides, *n*-C_7_ asphaltene has a lower H/C ratio (1.15) compared with resin II (1.32) because of the strong aromaticity of the former and its unsaturation degree.

The nanocatalyst was synthesized through the impregnation of salt precursors NiCl_2_∙6H_2_O (Merck, KGaA, Darmstadt, Germany) and Pd(NO_3_)_2_∙2H_2_O (Merck, KGaA, Darmstadt, Germany) following the incipient wetness method [31] over a CeO_2_ nanoparticulated support (Nanostructures & Amorphous Materials, Houston, TX, USA). The average particle size and surface area of the support were 21 nm and 65 m^2^∙g^−1^. The nanocatalyst was designed for final loads of Ni and Pd of 0.89 and 1.1 wt.%, respectively and is labeled as CeNi0.89Pd1.1 in accordance with a previous study on material optimization [16,17]. The catalyst presents a crystal size of 5.53–3.61 nm with dispersion of 25% and 36% for Ni and Pd crystals.

The nanofluid was prepared using 2000 mg∙L^−1^ of the CeNi0.89Pd1.1 nanocatalyst, 0.3 wt.% of Tween 80 surfactant (Panreac, Barcelona, Spain), and 0.2 wt.% of NaCl. The porous medium was composed of Ottawa-silica sand (Minercol S.A., Bogotá, Colombia) and the cleaning was done using methanol (99.8%, Panreac, Barcelona, Spain), hydrochloric acid (HCl, 37%, Panreac, Barcelona, Spain), and toluene (99.8%, Panreac, Barcelona, Spain), following the procedure detailed in a previous study [32].

### 2.2. Methods

#### 2.2.1. Adsorption Experiments

Oil model solutions of resin II and/or *n*-C_7_ asphaltenes in toluene were prepared at different concentrations between 100 and 2000 mg·L^−1^. The adsorption isotherms were constructed at 25 °C using 100 mg of functionalized and non-functionalized nanoparticles per 10 mL of solution. The mixtures were left under magnetic stirring at 300 rpm for 24 h until the system reached the adsorption equilibrium [33]. The nanoparticles with resin II and/or *n*-C_7_ asphaltene molecules adsorbed were separated by centrifugation at 4500 rpm for 45 min and the precipitate was analyzed using a Q50 thermogravimetric analyzer (TA Instruments Inc., New Castel, DE, USA) and corroborated through UV-vis measurements [34,35].

For the best system in terms of the adsorption and catalysis of the heavy oil fractions, adsorption isotherms for a solution model were constructed for the different resin to asphaltene ratios (R:A) of 8:2, 1:1, and 2:8. Regarding the quantity of *n*-C_7_ asphaltenes and resin II adsorbed on the nanoparticle for the composite systems, it was measured by combining the tests of the softening point (SP) and thermogravimetric analysis (TGA) based on our previous work [36]. Following the ASTM E28-12 standard [37], experiments to determine the SP were developed. The calibration curve SP vs. % *n*-C_7_ asphaltenes in the mixtures is shown in Figure S1 of the Supplementary Material. The amount of resin II or *n*-C_7_ asphaltenes adsorbed individually or in the mixture systems was calculated following the protocol detailed by Lozano et al. [36].

#### 2.2.2. Thermogravimetric Analyses

The catalytic steam gasification of adsorbed compounds on the nanoparticles was carried out using a Q50 thermogravimetric analyzer (TA Instruments, Inc., New Castel, DE, USA). The N_2_ flow was fixed at 100 mL·min^−1^ and H_2_O_(g)_ was introduced using a gas saturator filled with distilled water and controlled by a thermostatic bath with a flow rate of 6.30 mL·min^−1^ [38]. Finally, the amount of adsorbed asphaltenes and/or resins was set at 0.2 ± 0.02 mg·m^−2^ for all samples [28]. The samples were subjected to non-isothermal and isothermal procedures. Under non-isothermal conditions, the samples were subjected to temperatures between 100 and 600 °C at a heating rate of 20 °C·min^−1^. For the test under isothermal conditions, the samples were heated at 210, 220, and 230 °C for *n*-C_7_ asphaltenes and resins II adsorbed over nanoparticles and 360, 370, and 380 °C for the heavy fractions in the absence of nanoparticles.

#### 2.2.3. Oil Recovery

**Figure 1:**
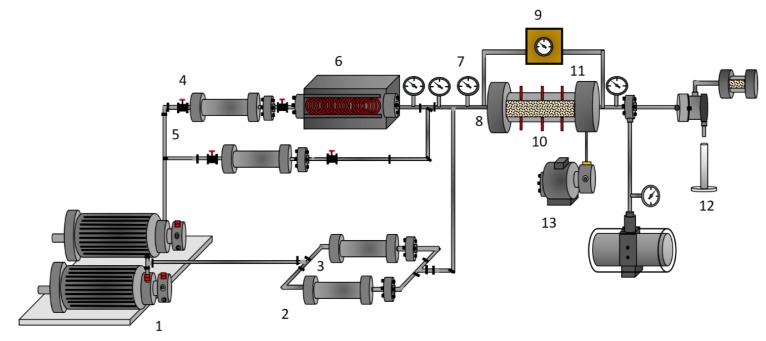
Experimental setup: (1) positive displacement pumps, (2,3,4,5) oil, brine water, and nanofluid-containing cylinders, respectively, (6) steam generator, (7) manometers, (8) thermocouple, (9) pressure transducer, (10) core holder, (11) sand packed bed, (12) sample output and (13) hydraulic pump.

The displacement test was divided into four main stages to recreate the injection conditions of the steam stimulation processes and evaluate the effect of the CeNi0.89Pd1.1 nanocatalyst in oil recovery and in situ upgrading. Table 2 shows the properties of the porous medium employed. Figure 1 shows a schematic representation of the experimental setup. The system consists of two positive displacement pumps (DB Robinson Group, Edmonton, AB, Canada), one to control the injection of oil and water and another to control the injection of water to the steam generator and nanofluid to the porous medium. The system also includes four cylinders (Max Servicios, S.A.S., Medellín, Colombia) containing EHO, synthetic brine, deionized water, and nanofluid; a steam generator (Thermo Scientific Waltham, MA, USA), thermocouples (Termocuplas, S.A.S., Medellín, Colombia); valves (Swagelok, Cleveland, OH, USA); manometers (Rosemount, Emerson, Chesterfield, MO, USA); fraction collectors; a pressure transducer (Rosemount, Emerson, Chesterfield, MO, USA); a hydraulic pump (Enercap, Actuant Corporation, Milwaukee, WI, USA); and a pressure multiplier. A leak test was performed by pressurizing the core-holder with N_2_ up to 1000 psi, where a change in 1% of pressure per hour was considered to be the maximum pressure reduction allowed during the leak test.

Before the steam injection, measurements of the absolute permeability (K) and the effective permeabilities of water (kw) and oil (ko) were performed. For this, 10 VP of each phase was injected in the order water–oil–water until a constant pressure drop was observed during each injection. The values of K, kw, and ko were calculated using the Darcy equation [39]. For this scenario, the system temperature was maintained at 80 °C, and the pore and overburden pressure were fixed at 150 and 800 psi, respectively.

For the construction of initial oil recovery by steam injection, 7 porous volumes of water equivalent (PVWE) were injected at 210 °C. Steam was injected between 3 and 5 mL·min^−1^ to prevent condensation into the coil line and porous medium. Under these conditions, steam was injected at a quality of 70%, achieved by the combination of a massive flow of steam and liquid water. The steam quality was calculated through numerical simulation of the steam generator and injector system and material balance between the points located at the exit of the steam generator and the entrance to the porous medium. Considering the steam injection conditions (*T* = 210 °C, *x* = 70%), the properties of the line connecting the points for the material balance (length = 0.63 m, thermal conductivity = 16.3 W·m^−1^·K^−1^, internal radius = 0.0046 m, and external radius = 0.00635 m) and using the Antoine correlation, heat losses, and the pressure at each node necessary to ensure the quality of steam at the entrance of the porous medium were calculated. Radial heat persistence was not considered because of the liquid–vapor equilibrium conditions of the system. The heat transfer coefficient was very high and therefore the resistance value could be neglected [40].

The injection of the nanocatalyst-based nanofluid was performed through the continuous nanofluid addition to the steam current for approximately 7 PVWE. The nanofluid was injected into the steam generator outlet at a rate between 0.5 and 1 mL∙min^−1^ before entering the porous medium. The pressure profile of the system was monitored throughout the displacement test to ensure the nanofluid transport in the steam stream. The oil recovery was obtained until no more oil was produced. Then, the porous media was left to stand for 12 h, and steam in the absence of the nanofluid was injected again until there was no oil production.

#### 2.2.4. Oil Characterization before and after Upgrading

The effectiveness of the CeNi0.89Pd1.1 nanoparticles in changing the physicochemical properties of the EHO was evaluated through API gravity, oil viscosity, and the *n*-C_7_ asphaltene content after each stage of the protocol described in the previous section. The API gravity measurements were performed using an Anton Paar Stabinger SVM 3000 (Madrid, Spain). The asphaltene content was calculated by micro deasphalting, following the protocol described in previous works [18,41]. The rheological behavior of the crude oil after each stage was evaluated through a Kinexus Pro (Malvern Instruments, Worcestershire, UK) rheometer, using a parallel plate-plate geometry and 0.3 mm of GAP at 25 °C and varying the shear rate from 0 to 100 s^−1^. The degree of viscosity reduction (DVR) was calculated using the viscosity of the untreated EHO ($\mu_{v,EHO}$) and upgraded crude oil ($\mu_{treated}$) after the three stages, using the following expression:

(1) $$DVR=\frac{\left(\mu_{v,EHO}-\mu_{treated}\right)}{\mu_{v,EHO}}\times 100$$

## 3. Modeling

### 3.1. Adsorption Model

The solid–liquid equilibrium (SLE) model [32,42] describes the adsorption isotherms of the asphaltenes and/or resins II over the nanocatalysts employed in this study, from the theory of adsorption and association of molecules in microporous surfaces, according the following relations:

(2) $$C_{E}=\;\frac{\psi H}{1+K\psi }e^{\left(\frac{\psi }{q_{m}\cdot SA\;}\right)}$$

(3) $$K=\;\frac{K_{T}RT}{A}$$

(4) $$\psi =\;\frac{-1+\sqrt{1+4K\xi }}{2K}$$

where, $C_{E}$(mg·g^−1^) is the concentration of asphaltenes and/or resins in the equilibrium, H(mg·g^−1^) refers to the affinity that the adsorbate presents for the adsorbent, K(g·g^−1^) is a parameter that indicates the self-association of *n*-C_7_ asphaltenes and/or resin II molecules on the surface of the nanoparticles, and $q_{m}$(g·g^−1^) is the maximum adsorption capacity of the nanocatalysts [42]. Finally, SA is the surface area of the catalyst, and ψ and ξ are defined as the adjustment parameters [42].

### 3.2. Effective Activation Energy Estimation

The activation energy was calculated from the following equation [43]:

(5) $$\frac{d\alpha }{dt}=K_{\alpha }\exp\left(-\frac{E_{\alpha }}{RT}\right)f\left(\alpha \right)$$

where $K_{\alpha }$(s^−1^) is a pre-exponential factor, R(J·mol^−1^·K^−1^) is the ideal gas constant, and $E_{\alpha }$ (kJ·mol^−1^) is the activation energy. On the other hand, dα/dt refers to the change in the conversion (α) of asphaltenes and/or resin II at a determined time during the decomposition reactions.

According to the isothermal conditions of operation, the integration of Equation (5) leads to:

(6) $$g(\alpha )=K_{\alpha }\exp\left(-\frac{E_{\alpha }}{RT}\right)t$$

Applying the natural logarithm on both sides of Equation (6), this can be written in linear terms as:

(7) $$ln\left(t_{a,i}\right)=ln\left(\frac{g\left(\alpha \right)}{K_{\alpha }}\right)+\frac{E_{\alpha }}{RTi}$$

Finally, by graphing $\ln(t_{a,i})$ vs. $1/T_{i}$ and obtaining the value of the slope, the value of the activation energy can be obtained.

### 3.3. Rheological Model

To describe the EHO rheological behavior in the different stages, the Cross model was employed:

(8) $$\mu =\mu_{\infty ,\gamma }\frac{\mu_{0,\gamma }-\mu_{\infty ,\gamma }}{1+{\left(\alpha_{c}\gamma \right)}^{m}}$$

where, μ (cP) is the fluid viscosity, m is a constant that represents the trend of a fluid to have a Newtonian behavior, $\alpha_{c}$(s) is the relaxation time, and $\mu_{0,\gamma }$ and $\mu_{\infty ,\gamma }$(cP) are the viscosities at zero shear rate and infinite shear rate, respectively.

## 4. Results and Discussion

### 4.1. Adsorption Isotherms

**Figure 2:**
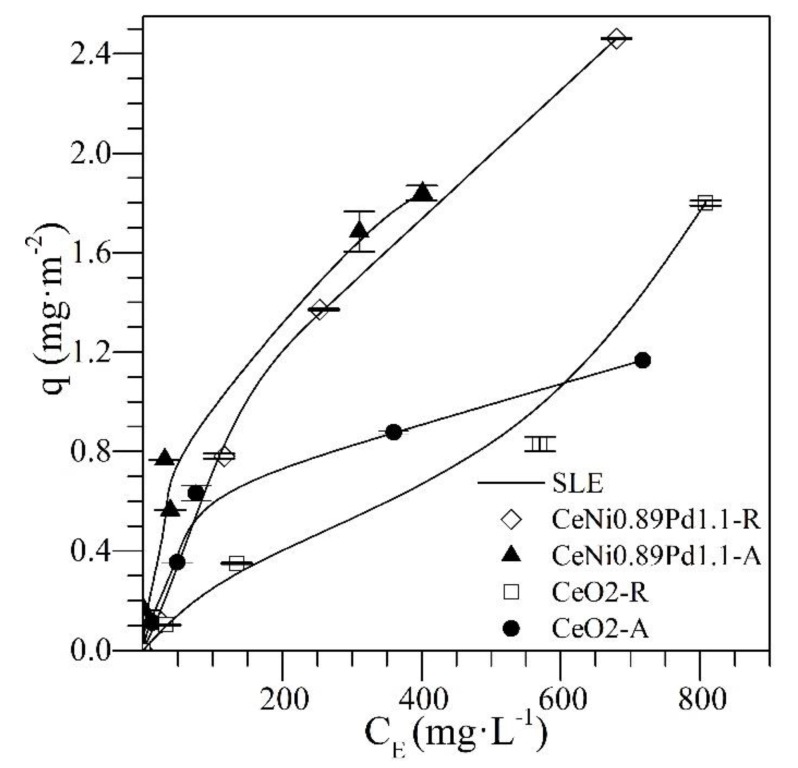
Adsorption isotherms of *n*-C_7_ asphaltenes (A) and resin II (R) onto CeNi0.89Pd1.1 and CeO_2_ nanoparticles evaluated at 25 °C. The solid lines are from the solid–liquid equilibrium (SLE) model and the symbols are experimental data.

The adsorption isotherms of resin II and *n*-C_7_ asphaltenes at 25 °C for the CeNi0.89Pd1.1 and CeO_2_ nanoparticles together with the SLE model fitting are shown in Figure 2. A high affinity can be observed between the *n*-C_7_ asphaltenes and the different nanoparticles used. The shape of the adsorption isotherms in the two systems is Type Ib according to the International Union of Pure and Applied Chemistry (IUPAC) [44]. This type of isotherm indicates a strong affinity between the adsorbate and the adsorbent. This property is always higher for the CeNi0.89Pd1.1 than for the CeO_2_ nanoparticles, indicating an increase in the adsorptive capacity of the nanomaterial with the addition of the transition element oxides (TEO) on its surface [45] through the formation of coordinated bonds between the heteroatoms and the functional groups of the transition elements [46].

**Figure 3:**
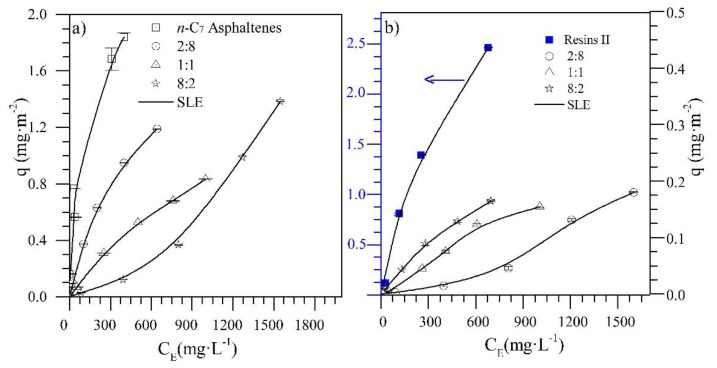
Adsorption isotherms of (**a**) *n*-C_7_ asphaltenes (A) and (**b**) resins II (R) on CeNi0.89Pd1.1 nanoparticles evaluated at 25 °C and from different ratios of R:A of 8:2, 1:1, and 2:8. The solid lines are from the SLE model and the symbols are experimental data.

Regarding the adsorption isotherms of resin II onto CeNi0.89Pd1.1 nanoparticles, a high affinity is observed over the range of concentrations evaluated. This behavior, according to the International Union of Pure and Applied Chemistry (IUPAC), corresponds to a Type I isotherm [44]. This result is due to the interactions formed by the functional groups of NiO and PdO on the nanoparticle surface with the active bonds and heteroatoms in the chemical structures of the resins [47]. Regarding the adsorption of resin II on the non-functionalized support, a type II isotherm is obtained [44]. When the concentration of resin II increases, molecules with a larger size have reduced diffusion through the material surface, leading to a high self-association around the nanoparticles’ active sites [48]. This indicates that, although the resin–resin interactions are low in the bulk phase, once adsorbed on the nanoparticle surface, self-association occurs through coordinated bonds and bonds between carbon-based molecules and heteroatoms (C–C, C–N, C–O) because of their polar character [49,50]. In this sense, for both resins and asphaltenes, the adsorption is higher for CeNi089Pd1.1 nanoparticles than for the support without TEOs. Because of the presence of transition elements, the adsorption of polar molecules on the nanoparticles is controlled to a high degree by the Lewis acidity [51], which favors the adsorption on the functionalized nanoparticles compared to the support. The nanoparticle that showed the best performance by adsorbing resin II and *n*-C_7_ asphaltenes was CeNi0.89Pd1.1. Therefore, the competitive adsorption between *n*-C_7_ asphaltenes and resin II for different R:A ratio was evaluated with this sample, and the results are shown in Figure 3a,b together with the SLE model fitting.

Figure 3a shows that the adsorption affinity between *n*-C_7_ asphaltenes and CeNi0.89Pd1.1 nanoparticles decreases as the amount of resin II in the system increases. For the R:A ratios of 2:8 and 1:1, the adsorption isotherms are also Type I, and for the 8:2 system, the isotherm obtained behaves as type III according to the IUPAC [44]. The change in the adsorbate–adsorbent affinity may be related to an interruption or change in the colloidal structure of *n*-C_7_ asphaltenes because of the presence of resins that prevents asphaltene–asphaltene interactions, thus avoiding self-association [52,53]. Also, because of the high selectivity that CeNi0.89Pd1.1 nanoparticles present for resin II, competitive adsorption between the two polar fractions may be generated. In this way, there is a reduction in the active sites on the surface of the nanoparticles occupied by resin. These results are consistent with those published by Lozano et al. [36] and Franco et al. [26], where the adsorption of asphaltenes on nanoparticles of different chemical nature is affected to a small extent by the presence of resin I in the system.

Figure 3b shows the adsorption isotherms for resin II in the presence of *n*-C_7_ asphaltenes at different R:A ratios. In general, it can be seen that the adsorption isotherms keep their type I behavior for the ratios 8:2 and 1:1. However, for the 2:8 system, a high presence of *n*-C_7_ asphaltenes generates a change in the type of isotherm to Type III. In both cases, it is indicated that the adsorption of resin II is also influenced by the presence of *n*-C_7_ asphaltenes. The adsorption of resins II over the nanoparticles increases as the amount of *n*-C_7_ asphaltenes in the system decreases, following the order 2:8 < 1:1 < 8:2. The interactions formed between the functional groups of the metal oxides and the heteroatoms in the molecular structure of resins II are stronger than the resin–resin and resin–asphaltene interactions because of the presence of acidic and basic sites that the TEO and CeO_2_ generate [54]. The multilayer behavior of the 2:8 ratio could be due to the resin–asphaltene interactions being more significant than the resin–resin interaction [55].

Table 3 shows the H, K, y, and $Q_{m}$ parameters of the SLE model for the adsorption of asphaltenes and resin II in different R:A ratios over the functionalized and CeO_2_ nanoparticles. According to the *H* parameter, there is greater affinity for CeNi0.89Pd1.1 material with the resin II and *n*-C_7_ asphaltenes as individual components. For the adsorption isotherms of *n*-C_7_ asphaltene in the presence of resin II, the H parameter increased as the amount of resin II in the system increased because of the high selectivity that CeNi0.89Pd1.1 nanoparticles presented for resin II. In this way, it appears that the preference of *n*-C_7_ asphaltenes to be present in the adsorbed phase is reduced by the presence of resin II. Concerning the degree of self-association of *n*-C_7_ asphaltenes on the surface of the nanoparticles, the K parameter follows the R:A trend of 2:8 < 1:1 < 8:2, which indicates that the asphaltene–asphaltene interactions are reduced because of the presence of stronger asphaltene–resin interactions [55,56]. Also, the value of H for the adsorption of resin II decreases as the amount of *n*-C_7_ asphaltene increases, and the K parameter behaves similarly to the self-association of *n*-C_7_ asphaltenes. This is also due to the fact that resin–resin interactions are affected by asphaltene–resin interactions [57].

### 4.2. Prediction of the Adsorbed Amount of n-C_7_ Asphaltenes

For a better understanding of the influence of resin II on the adsorption phenomenon of *n*-C_7_ asphaltenes over the CeNi0.89Pd1.1 nanoparticles, the adsorbed amount of asphaltenes was predicted as individual systems for the different R:A ratios employed. If the behavior of *n*-C_7_ asphaltenes were affected by the amount of resin II in the systems, there would be a difference between the predicted and the experimental adsorbed amounts. On the other hand, if the resin had no effect on the asphaltene adsorption, the adsorption of the latter would depend only on its concentration and the occupation of the active sites of the nanoparticle by resin II. For the prediction of the adsorbed amount of *n*-C_7_ asphaltenes ($q_{predicted}$), it was assumed that for a fixed R:A system, the amount adsorbed would correspond to a fraction of the amount of individual asphaltenes adsorbed. That is, for a 1:1 system, the amount of adsorbed *n*-C_7_ asphaltenes corresponds to 50% of the mass fraction initially adsorbed. A linear plot $q_{predicted}$ versus $q_{experimental}$ that represents a good prediction would imply a slope of m= 1 and an intercept of b = 0 associated with $R^{2}$ = 1.0, indicating that asphaltene adsorption depends mainly on the amount of asphaltenes present in the system.

**Figure 4:**
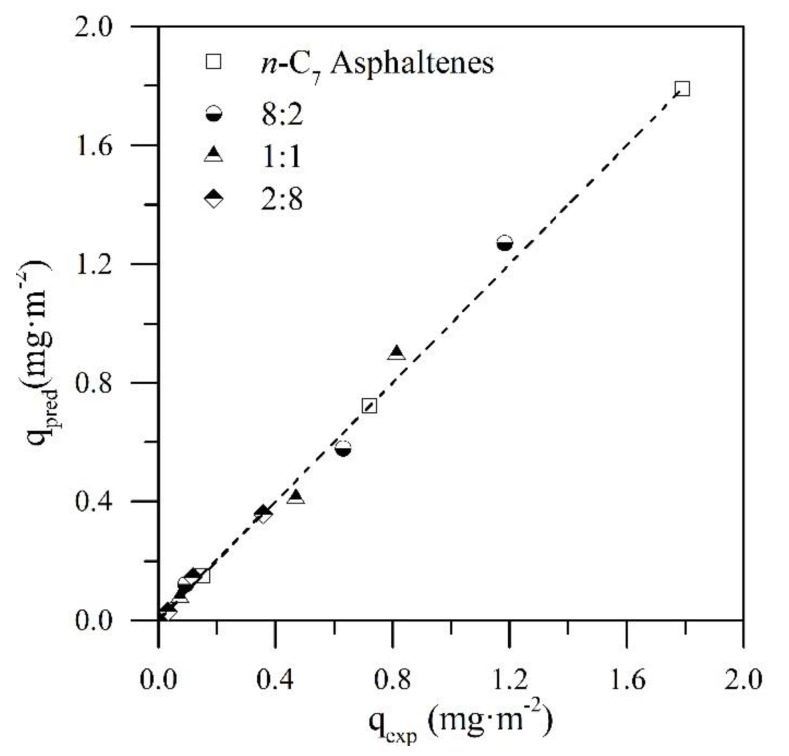
Linear plots of *q_predicted_* as a function of *q_experimental_* for the adsorption of *n*-C_7_ asphaltenes (A) over CeNi0.89Pd1.1 nanoparticles in the presence of resin II (R) in the systems at different R:A ratios of 2:8, 1:1, and 8:2.

Figure 4 shows the prediction plot of the adsorbed amount of *n*-C_7_ asphaltenes in the CeNi0.89Pd1.1 nanoparticles in the absence and presence of resin II. In addition, Table 4 shows the values of the slope and the intercept of the equation associated with the linear fit and the respective errors. According to Figure 4 and the data in Table 4, a very accurate prediction was observed for the adsorbed amount of *n*-C_7_ asphaltenes on CeNi0.89Pd1.1 nanoparticles in the presence of resin II for the R:A systems evaluated. The values of the slopes in all cases were close to 1, and the intercepts were close to the origin with $R^{2}$ > 0.98. These results indicate that the adsorption of *n*-C_7_ asphaltenes is controlled mainly by the concentration of these in the system and by the affinity that the nanoparticle presents for resin II. These results are in agreement with those reported by Franco et al. [26].

### 4.3. Thermogravimetric Experiments

**Figure 5:**
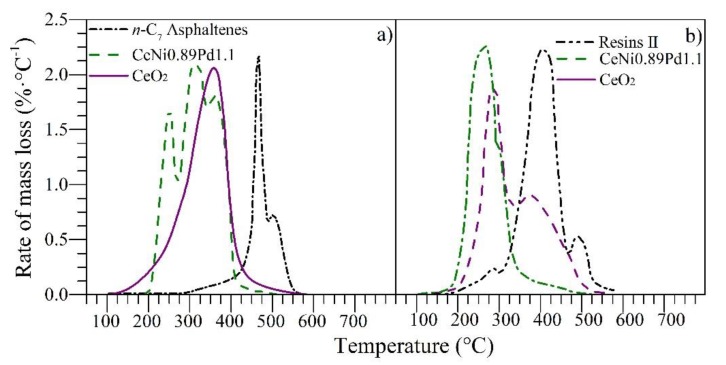
Rate of mass loss as a function of temperature for steam gasification of (**a**) *n*-C_7_ asphaltenes and (**b**) resin II in the absence and presence of CeO_2_ and CeNi0.89Pd1.1 nanoparticles. Panel (a) was adapted from Medina et al. [16].

The nanoparticles evaluated in this work were tested in an N_2_ atmosphere saturated with H_2_O_(g)_ for the catalytic steam gasification of *n*-C_7_ asphaltenes and resin II. Figure 5 shows the rate of mass loss for *n*-C_7_ asphaltenes and resins II in panels a and b, respectively. It can be observed from Figure 5 that for virgin resin II and *n*-C_7_ asphaltenes, i.e., before adsorption onto nanoparticles, the decomposition occurs at 420 and 450 °C, respectively. Nevertheless, when they are adsorbed on the nanoparticles, their decomposition occurs at lower temperatures.

Functionalized and non-functionalized nanoparticles reduce the main decomposition peak from 400 to 220 and 270 °C for resin II and from 450 to 220 and 370 °C for *n*-C_7_ asphaltenes, respectively. However, the heavy oil fractions do not decompose completely at these temperatures, and therefore, the conversion continues at higher temperatures. This result suggests the presence of high, medium, and low molecular weight sizes that vary from alkyl chains that decompose at low temperatures (<250 °C) to polycyclic aromatic hydrocarbons (PAH), whose decomposition is achieved at average temperatures of around 450 °C.

Besides, the functionalized nanoparticle manages to reduce the resin II decomposition temperature to 220 °C, which inhibits the development of additional reactions of resin II after its initial cracking, showing a single peak of decomposition [16]. The catalytic effect was improved with the addition of oxides of transition elements on the CeO_2_ surface for *n*-C_7_ asphaltene and resin II decomposition. The good performance of the functionalized nanoparticles is due to the ability of the support to interact with the nanocrystals to produce and promote cracking and isomerization reactions [58]. The Ce–Ni interactions are able to promote the production of the water–gas shift reaction through vacancies of oxygen anions provided by the CeO_2_ [20]. Meanwhile, the interactions of the support with noble elements like Pd [22] promote the production of hydrogen and oxygen which controls the stability of highly polar molecules. Hence, further experiments under isothermal conditions were performed with the functionalized nanoparticles and for different R:A ratios.

**Figure 6:**
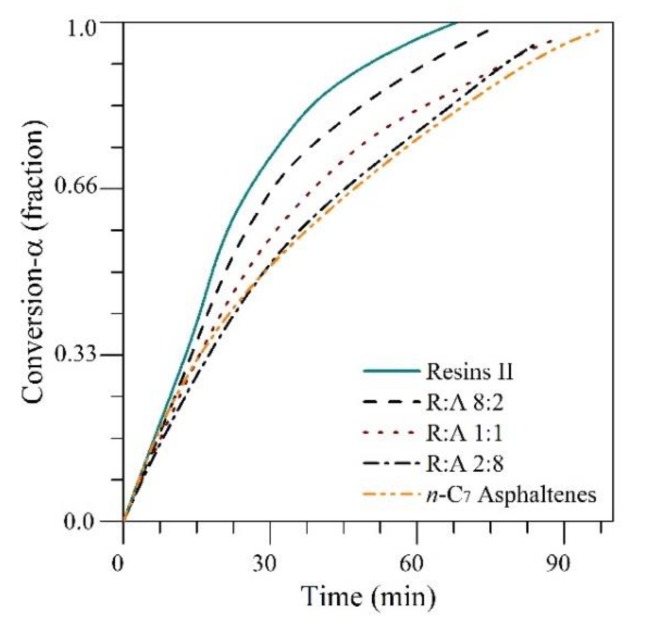
Isothermal conversion times at different R:A ratios of 8:2, 1:1, and 2:8 in the presence of CeNi0.89Pd1.1 nanoparticles at 220 °C.

Figure 6 shows the isothermal conversions at 220 °C for different R:A ratios of 8:2, 1:1, and 2:8 and for the individual components. The conversion of resin II at any time is always greater than the conversion of asphaltenes. In addition, the conversion of the R:A systems decreases as the amount of *n*-C_7_ asphaltenes in the mixture increases because of the high presence of heteroatoms and metals as well as the high refractory behavior of *n*-C_7_ asphaltenes.

### 4.4. Effective Activation Energy

For the effective activation energy estimation, a plot of $\ln(t_{a,i})$ vs. $1/T_{i}$ was obtained using the conversion of each fraction at three different temperatures. The value of the activation energy was obtained from the slope. The isothermal conversions for resin II and *n*-C_7_ asphaltenes in the presence of nanoparticles were made at 210, 220, and 230 °C, and for the fraction in the absence of nanoparticles, the employed temperatures were 360, 370, and 380 °C. These curves are shown in Figures S2–S4 of the Supplementary Materials.

**Figure 7:**
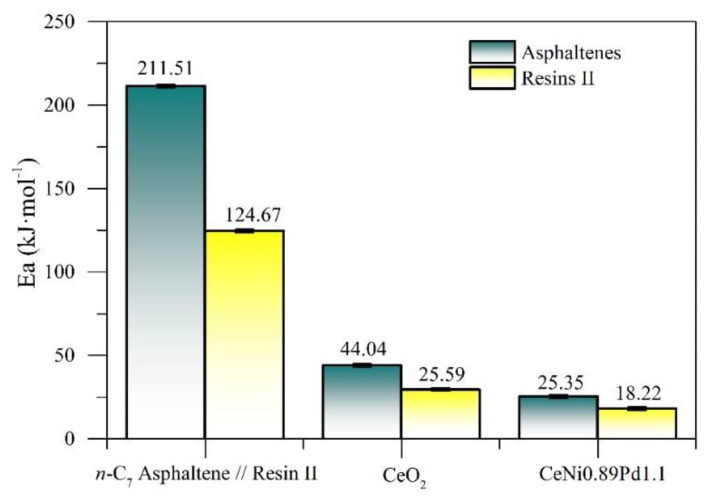
Estimated effective activation energy ($E_{a}$) for isothermal gasification of resin II with CeO_2_ and CeNi0.89Pd1.1 nanoparticles for temperatures between 230 and 250 °C and without nanoparticles for temperatures between 360 and 380 °C.

Figure 7 shows the activation energy values for the resin II and *n*-C_7_ asphaltene decomposition in the presence and absence of the functionalized nanoparticles and the CeO_2_ support. There is a significant decrease in the activation energy values for resin II adsorbed on the different nanoparticles, with the CeNi0.89Pd1.1 system being the one that generates the biggest decrease in $E_{a}$. The presence of oxides on the surface of the nanoparticle generates a change in the decomposition mechanism of the heavy oil fractions, and their performance depends, to a considerable extent, on the chemical nature of the nanoparticle. In addition, the activation energy values are lower for the decomposition of resin II than for *n*-C_7_ asphaltenes.

**Figure 8:**
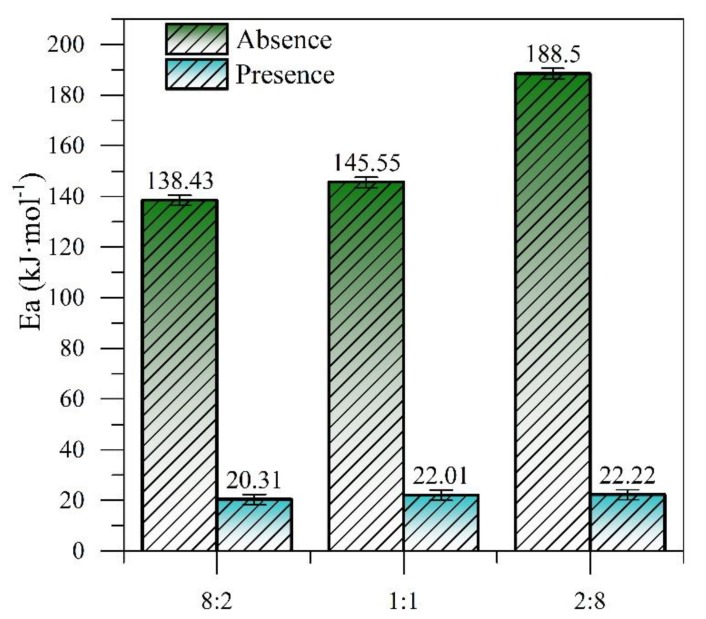
The estimated effective activation energy for isothermal catalytic gasification of the R:A systems in the absence and presence of CeNi0.89Pd1.1 nanoparticles for temperatures between 230 and 250 °C and without nanoparticles for temperatures between 360 and 380 °C.

The activation energy values for the R:A systems in the presence and absence of CeNi0.89Pd1.1 nanoparticles were calculated by following the same procedure and the results are shown in Figure 8. The required activation energy increases as the amount of asphaltenes in the system increases, both in the presence and absence of the CeNi0.89Pd1.1 nanoparticles. Nevertheless, the nanoparticles reduce the activation energy values necessary to carry out the decomposition reactions of the systems to 21.51 ± 1.05 kJ∙mol^−1^ independently of the amount of asphaltenes in the system.

Cerium oxide nanoparticles have the ability to regenerate their Ce^4+^ atoms through a redox mechanism, maintaining the catalytic power of the support. This is achieved by carrying out processes of adsorption of reagents (H_2_O and heavy oil fractions), decomposition and chemical change of adsorbed molecules, desorption of reaction products between the support and reagents (CO_2_, CH_4_, LHD, CO, among others), and the interactions between the products and the active sites of the nanocatalyst. Here, the chemical reactions between the hydrocarbons Equation (9) and carbon monoxide Equation (10) with CeO_2_ to produce Ce^3+^ ions are shown, where $V_{o}^{\cdot \cdot }$ is the oxygen vacancy produced from the redox cycle Ce^4+^/Ce^3+^:

(9) CeO2+HmCn→12xVo··+ Ce1-x4++ Cex3++ O2−12x+ H2O + CO2

(10) CeO2 + CO→12xVo·· + Ce1−x4+ + Cex3++O2−12x + CO2

**Figure 9:**
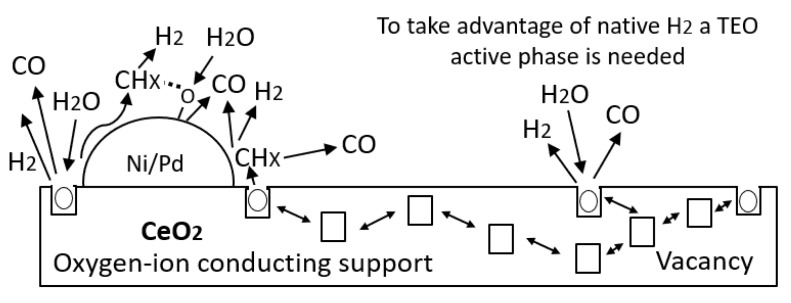
Graphical representation of oxygen ion vacancy conducting support to active sites of transition element oxides of Ni and Pd for hydrogen, methane, dioxide carbon, and monoxide carbon production.

In this way, the reduction of Ce^4+^ to Ce^3+^ leads to the production of water vapor molecules and CO_2_. Then, through a redox cycle, the initial oxidation state is recovered and the decomposition of asphaltenes and resins continues. However, the high performance of this catalyst is also associated with the active phases formed by the TEO on the surface of the support. The resulting species –O and –OH from the dissociative adsorption of water by the lower valence state of cerium may be transferred to nickel and palladium and react with surface carbonaceous species. Besides, through the movement of oxygen vacancies formed by the change in the oxidation state of the CeO_2±δ_ species and the destabilization of the same, the reagents are transferred to the active sites of the transition element oxides, as schematized in Figure 9.

### 4.5. Dynamic Tests of Oil Recovery

**Figure 10:**
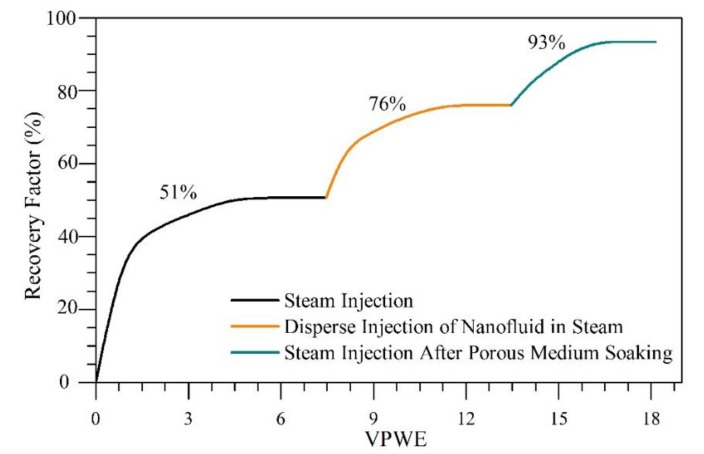
Oil recovery curve for steam injection assisted by CeNi0.89Pd1.1 nanocatalyst dispersed in the steam stream during the stages: (1) continuous steam injection, (2) dispersed injection of nanofluid in steam, and (3) steam injection after porous medium soaking.

The oil recovery curve for the dispersed injection of CeNi0.89Pd1.1-based nanofluid in the steam stream to assist the steam injection technology is shown in Figure 10. In addition, the saturation states of both fluids, water and oil, are shown in Table 5. For these tests, the effective permeabilities of water (kw) and oil (ko) were estimated to be 3744 and 2264 mD, respectively, which is in agreement with the properties of an HO reservoir. After the injection of 7 PVWE, an oil recovery of 51% was obtained because of the transfer of heat to the rock and fluids, promoting its thermal expansion. In addition, other related mechanisms are the possible gravitational segregation of fluids, the volatilization of the lightest hydrocarbons and the disaggregation of the viscoelastic network of the oil. After the dispersed injection of the nanofluid in the steam, an increment of 25% in the oil recovered was obtained. This is, to a great extent, due to the small size of the liquid droplets as they are carried by steam stream to achieve higher penetration. Finally, after soaking the porous medium for 12 h, steam was injected again, and an ultimate oil recovery of 93% was reached.

The good performance obtained in the test carried out through nanofluid injection dispersed by the steam stream compared with the batch injection reported in previous studies [9,18] is related to the rapid interaction between the water vapor and the active sites of the CeNi0.89Pd1.1 nanocatalyst [59]. Moreover, the rate of C–C bond cleavage was improved in the catalytic cracking of heavy oil fractions, and higher amounts of H_2_ and O_2_ species could be produced from the splitting of steam molecules to react with the asphaltenes and resins over the catalyst surfaces [59]. At the same time, the increase in asphaltene–CeO_2_ interactions enabled the enhanced adsorption and release of oxygen through the Ce^4+^/Ce^3+^ redox cycle [60]. Thus, the vacancies of oxygen anions on the surface of the nanocatalyst became unstable [21,61], cracking the heavy oil fractions via partial oxidation through the oxygen production promoted by the CeNi0.89Pd1.1 nanocatalyst or water via the redox reaction [59]. In addition, the redox reaction mechanism between the nanocatalyst, the heavy hydrocarbons, and the H_2_O_(g)_ results in the formation of hydrogen as a byproduct, which could participate in the stabilization of the previously cracked free radicals [62].

### 4.6. Crude Oil Characterization before and after Nanofluid Injection

**Figure 11:**
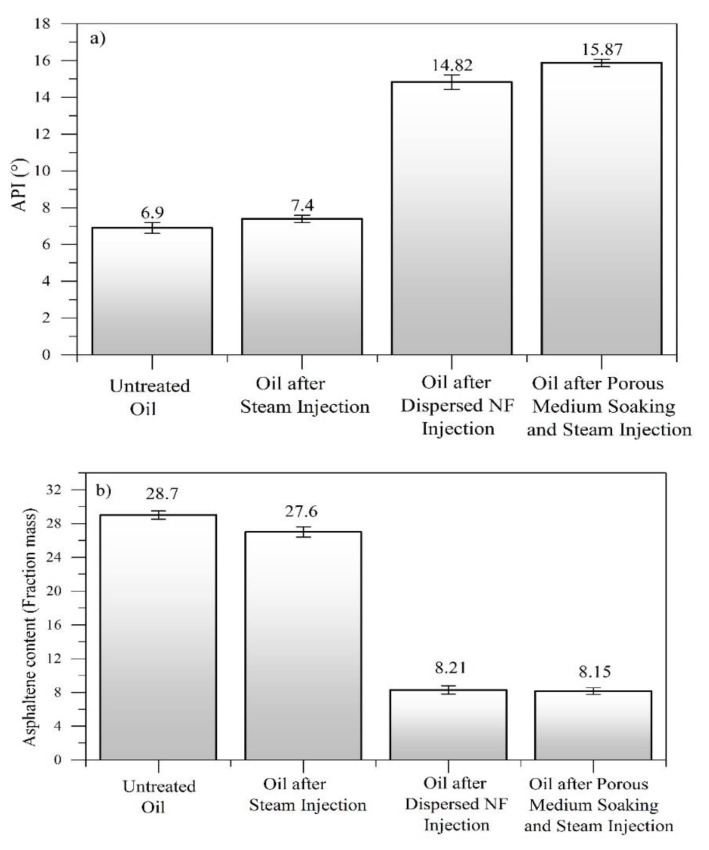
(**a**) API gravity and (**b**) asphaltene content in wt.% for untreated extra heavy oil and crude oil recovered after the steam injection, during the injection of CeNi0.89Pd1.1 nanocatalyst-based nanofluid dispersed in a steam stream, and after soaking for 12 h.

Panels a and b in Figure 11 show the values of API gravity and asphaltene content, respectively, for the untreated EHO, crude oil after steam injection, crude oil recovered by the dispersed injection of the nanofluid, and after a soaking time of 12 h. As observed, the API gravity increased and the asphaltene content reduced after the steam injection with the CeNi0.89Pd1.1 nanocatalyst. The results showed increases in API of 114.8% and 130% before and after the soaking treatment, confirming that the reaction time between adsorbed heavy fractions and nanocatalysts occurs immediately in the presence of steam. Furthermore, the asphaltene content was reduced by around 72% owing to the catalytic conversion of these compounds by the high catalytic activity of the nanoparticles employed to assist the technology.

**Figure 12:**
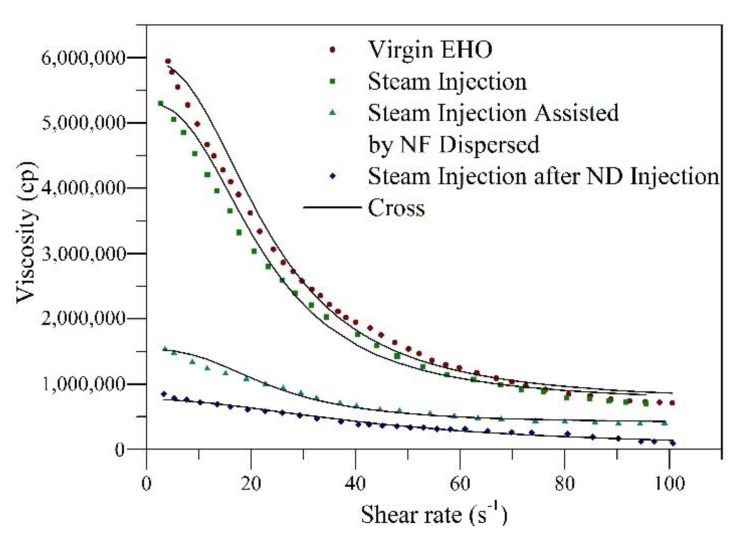
Rheological behavior at 25 °C and a shear rate from 0 to 100 s^−1^ for untreated extra heavy oil (EHO) and crude oil recovered after the steam injection, during the injection of CeNi0.89Pd1.1 nanocatalyst-based nanofluid in the steam stream, and after soaking for 12 h.

Rheological behavior experiments were conducted at 25 °C for oil samples taken after each step of the displacement test. Figure 12 shows the viscosity of the samples as a function of the shear rate together with the fit of the Cross model. Also, Table 6 summarizes the estimated rheological parameters found from the regression analysis. In Figure 12, it is observed for all samples that with the increase in shear rate, the viscosity of the crude oil decreases because of the breakdown of the internal structure of the crude oil, typical of shear-thinning pseudo-plastic fluids. With the injection of the steam without nanoparticles, a decrease in the heavy oil viscosity is appreciable, which could be related to the reduction in the cohesive forces on the molecular structures of combined asphaltene–resin compounds. For the scenarios assisted by nanotechnology, there were more pronounced reductions in viscosity, showing a higher decrease for the crude oil obtained after soaking for 12 h. The viscosity reduction mechanisms associated with the presence of the nanoparticles are the fragmentation and subsequent redistribution of the *n*-C_7_ aggregate asphaltenes [63], breaking the bonds and hindering the further formation of the heaviest compounds through the hydrogenation of free radicals and the asphaltene decomposition into lighter components. These results are in agreement with those reported by Franco et al. [9] and Cardona et al. [18].

The fitting of the Cross model presents an RMSE% < 1.0. The viscosity at zero ($\mu_{0,\gamma }$) and infinity shear rate ($\mu_{\infty ,\gamma }$) are consistent with the experimental data, following the order crude oil after steam injection after porous media soaking < crude oil during nanofluid dispersed injection in steam stream < crude oil after steam injection < EHO. Considering the values of m ≈ 1, the non-Newtonian behavior of the untreated oil is corroborated. These results are in agreement with those obtained by Franco et al. [9]. Finally, the degree of viscosity reduction (DVR) was calculated at a shear rate of 10 s^-1^ using Equation (1). The results obtained indicate viscosity reductions of 85%, 75%, and 10% for the crude oil recovered after the steam injection post soaking time during the nanocatalyst-based nanofluid injection by the steam stream and steam injection without nanoparticles, respectively. This reduction in viscosity could lead to a reduction in the costs associated with transport and refining operations, mitigation of the environmental impact through the naphtha consumption reduction, as well as an increase in the sale price for crude oil produced.

## 5. Conclusions

The present study shows, for the first time, the effect of resin II on the processes of adsorption and catalytic steam gasification of *n*-C_7_ asphaltene on functionalized and non-functionalized ceria nanoparticles. Among the most important results, functionalized nanoparticles achieved higher adsorption of both fractions as well as a higher decomposition temperature. The presence of resin did not affect the amount of asphaltene adsorption over the material. In addition, the increase in the resin content promoted a higher conversion in a shorter amount of time.

The dispersed injection of nanocatalyst-based nanofluid has been presented as a promising technique for assisting steam injection processes. In this case, the liquid drops are dragged by the steam stream, reaching a greater depth of penetration in the porous medium. In this way, a higher quantity of extra-heavy crude oil can be contacted and nanoparticles can be better distributed in the pore throats. An increase in oil recovery from 51% to 93% at the end of the displacement test was obtained. Also, the quality of crude oil was improved considerably in terms of the API gravity, asphaltene content, and viscosity. The API gravity increased from 6.9° to 15.87°. The asphaltene content reduced from 28.7 to 8.15 wt.%, thus obtaining a crude oil with a viscosity 85% lower than the untreated one.

## Figures and Tables

**Table 1:** Elemental composition of *n*-C_7_ asphaltenes and resin II of a Colombian extra-heavy crude oil.

	Elemental Mass Fraction Concentration (%)				
Fraction	Carbon	Hydrogen	Nitrogen	Oxygen	Sulphur
Resin II	82.10	9.00	0.54	2.26	6.10
*n*-C_7_ Asphaltenes	81.70	7.80	0.33	3.57	6.60

**Table 2:** Properties of porous media employed for the oil recovery experiments.

Property	Value
Mineralogy	99% Silica
Length	67 cm
Diameter	3.8 cm
Porosity	26.5%
Porous Volume	194 cm^3^
Initial oil saturation state	77.4%
Initial water saturation state	22.6%

**Table 3:** Estimated SLE model parameters of *n*-C_7_ asphaltenes and/or resin II adsorption onto different nanoparticles for different resin–asphaltene ratios (R:A ratios).

Sample		*n*-C_7_ Asphaltenes				Resin II			
	R:A	H (mg·g^−1^) × 10^−2^	K (g·g^−1^) × 10^−2^	q_m_ (mg·m^−2^)	% RSM	H (mg·g^−1^) × 10^−2^	K (g·g^−1^) × 10^−2^	q_m_ (mg·m^−2^)	% RSM
CeNi0.89Pd1.1	Individual	6.02	3.91	0.22	0.01	3.26	0.48	0.19	0.1
CeO_2_	Individual	10.12	20.7	0.12	0.02	4.03	7.62	18.57	0.1
CeNi0.89Pd1.1	2:8	6.70	4.32	0.16	0.01	1552	8.81	25.66	0.2
	1:1	10.61	4.66	0.11	0.02	985.1	7.66	0.22	0.1
	8:2	1343	5.77	27.17	0.01	3.33	2.33	0.25	0.2

**Table 4:** Estimated slopes and intercepts of the linear plots of *q_predicted_* as a function of *q_experimental_* for the adsorption of *n*-C_7_ asphaltenes (A) over CeNi0.89Pd1.1 nanoparticles in the presence of resin II (R) in the systems at different R:A ratios.

R:A Ratio	Slope	Intercept	$R^{2}$
2:8	1.04	0.007	0.99
1:1	1.06	−0.002	0.98
8:2	1.04	−0.007	0.99

**Table 5:** Oil (Sor) and water (Swr) residual saturation states in steam injection processes assisted by CeNi0.89Pd1.1 nanocatalyst dispersed in the steam stream.

Stage	S_wr_ (%)	S_or_ (%)
Steam Injection	62.3	24.0
Steam injection assisted by ND	81.5	14.0
Steam injection after ND injection	94.6	7.0

**Table 6:** Estimated rheological parameters by the Cross Model for virgin extra heavy oil (EHO) and crude oil recovered after the steam injection, during the injection of CeNi0.89Pd1.1 nanocatalyst-based nanofluid in the steam stream, and after soaking for 12 h.

Parameters	Virgin EHO	EHO after Steam Injection	EHO during Nanofluid Dispersed Injection	EHO Post Nanofluid Dispersed Injection
$\mu_{o,\gamma }$ **× 10^6^**	5.95	5.31	0.55	0.27
$\mu_{\infty ,\gamma }$ **× 10^6^**	7.10	7.04	1.60	0.98
$\alpha_{c}$ **× 10^−2^**	4.48	4.44	4.32	4.01
m	0.87	0.83	0.45	0.38
%RMSE	0.34	0.29	0.21	0.33
**DVR (%)**	-	10	75	85

## Supplementary Materials

The following are available online at https://www.mdpi.com/2079-4991/9/12/1755/s1. Figure S1. Softening point calibration curve. Figure S2. Isothermal conversion times at different temperatures for (a) virgin resin II and (b) virgin *n*-C_7_ asphaltenes at 360, 370, and 380 °C. Figure S3. Isothermal conversion times at different temperatures for *n*-C_7_ asphaltenes adsorbed onto (a) CeNi0.89Pd1.1 and (b) CeO_2_, at 230, 240, and 250 °C. Figure S4. Isothermal conversion times at different temperatures for resins II adsorbed onto (a) CeNi0.89Pd1.1 and (b) CeO_2_, at 230, 240, and 250 °C.
