# Supplementary material for: Upgrading of Extra-Heavy Crude Oils by Dispersed Injection of NiO–PdO/CeO2±δ Nanocatalyst-Based Nanofluids in the Steam
Source: Nanomaterials (Basel). 2019 Dec 10;9(12):1755. doi: 10.3390/nano9121755 (PMC6956154; doi:10.3390/nano9121755)
Supplement: Supplementary file 1 [file nanomaterials-09-01755-s001.pdf]

nanomaterials

Supplementary Materials

Upgrading of Extra-Heavy Crude Oils by Dispersed Injection of NiO–PdO/$CeO_{2\pm\delta}$ Nanocatalyst-Based Nanofluids in the Steam

Oscar E. Medina [1], Cristina Caro-Vélez [2], Jaime Gallego [3], Farid B. Cortés [1], Sergio H. Lopera [2] and Camilo A. Franco [1,*]

1 Grupo de Investigación en Fenómenos de Superficie—Michael Polanyi, Departamento de Procesos y Energía, Facultad de Minas, Universidad Nacional de Colombia, Sede Medellín, Medellín 050034, Colombia; oemedinae@unal.edu.co (O.E.M.); fbcortes@unal.edu.co (F.B.C.)
2 Grupo de Yacimientos de Hidrocarburos, Departamento de Procesos y Energía, Facultad de Minas, Universidad Nacional de Colombia, Medellín 050034, Colombia; ccarov@unal.edu.co (C.C.-V.); shlopera@unal.edu.co (S.H.L.)
3 Química de Recursos Energéticos y Medio Ambiente, Instituto de Química, Universidad de Antioquia UdeA, Calle 70 No. 52–21, Medellín 050010, Colombia; andres.gallego@udea.edu.co
* Correspondence: caafrancoar@unal.edu.co

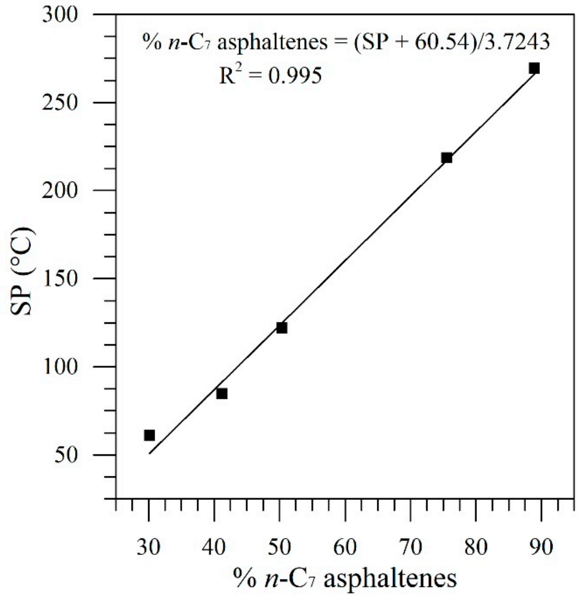

Figure S1. Softening point calibration curve.

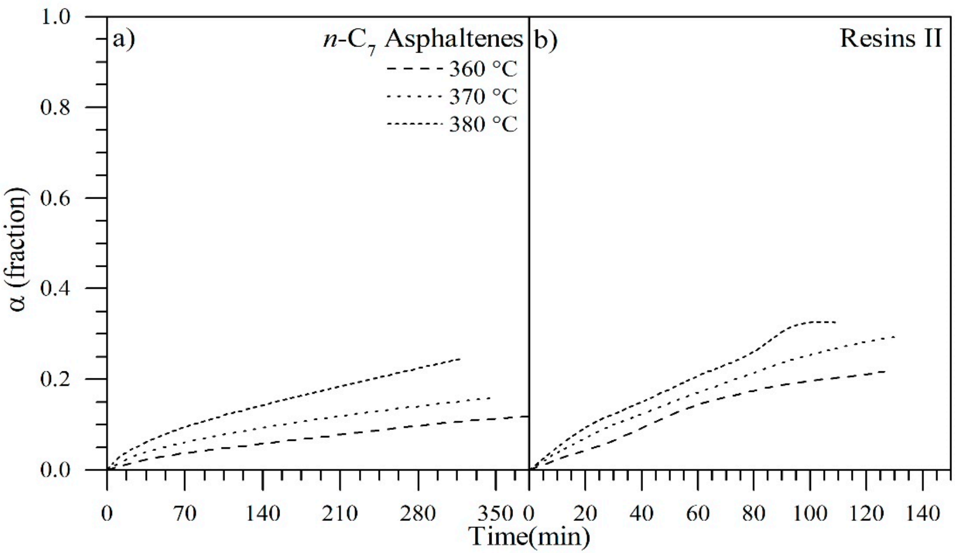

Figure S2. Isothermal conversion times at different temperatures for (**a**) virgin resin II and (**b**) virgin n-C_7 asphaltenes at 360, 370, and 380 °C.

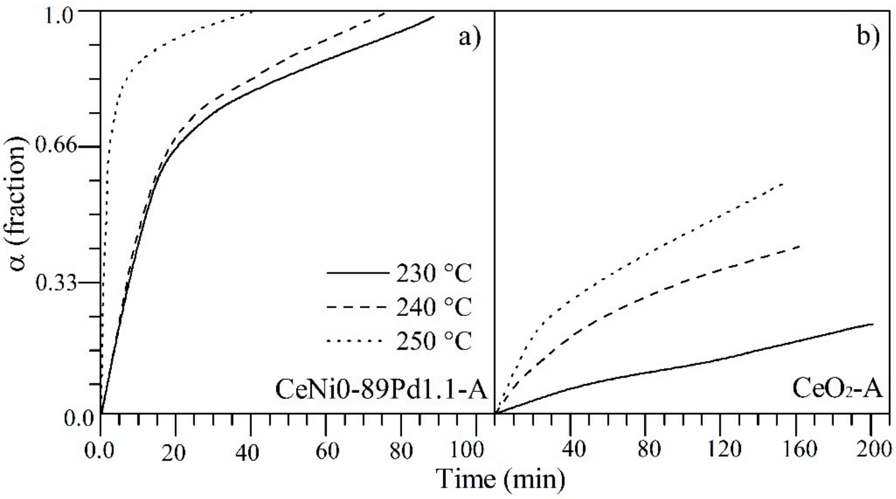

Figure S3. Isothermal conversion times at different temperatures for n-C_7 asphaltenes adsorbed onto (**a**) CeNi0.89Pd1.1 and (**b**) CeO_2, at 230, 240, and 250 °C. Isothermal conversion was taken from Medina et al. [1].

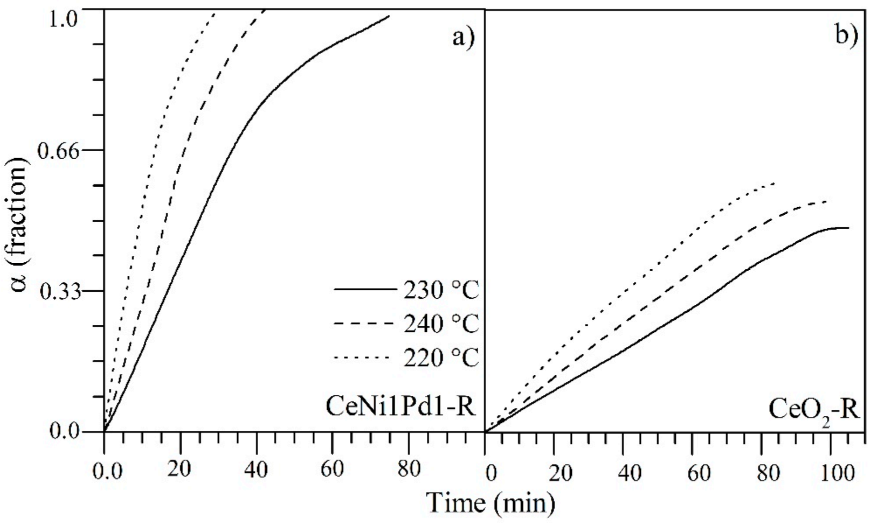

Figure S4. Isothermal conversion times at different temperatures for resins II adsorbed onto (**a**) CeNi0.89Pd1.1 and (**b**) CeO_2, at 230, 240, and 250 °C.

References.

1. Medina, O.E.; Gallego, J.; Arias-Madrid, D.; Cortés, F.B.; Franco, C.A. Optimization of the Load of Transition Metal Oxides (Fe2O3, Co3O4, NiO and/or PdO) onto CeO2 Nanoparticles in Catalytic Steam Decomposition of n-C7 Asphaltenes at Low Temperatures. *Nanomaterials* **2019**, *9*, 401.
